# Metabolomics Reveals Metabolic Targets and Biphasic Responses in Breast Cancer Cells Treated by Curcumin Alone and in Association with Docetaxel

**DOI:** 10.1371/journal.pone.0057971

**Published:** 2013-03-05

**Authors:** Mathilde Bayet-Robert, Daniel Morvan

**Affiliations:** 1 University of Auvergne-UDA, 49 boulevard François Mitterrand, F-63001 Clermont-Ferrand, France; 2 Comprehensive Cancer Centre Jean Perrin, 58 rue Montalembert, F-63011 Clermont-Ferrand, France; Wayne State University School of Medicine, United States of America

## Abstract

**Background:**

Curcumin (CUR) has deserved extensive research due to its anti-inflammatory properties, of interest in human diseases including cancer. However, pleiotropic even paradoxical responses of tumor cells have been reported, and the mechanisms of action of CUR remain uncompletely elucidated.

**Methodology/Principal Findings:**

^1^H-NMR spectroscopy-based metabolomics was applied to get novel insight into responses of MCF7 and MDA-MB-231 breast cancer cells to CUR alone, and MCF7 cells to CUR in cotreatment with docetaxel (DTX). In both cell types, a major target of CUR was glutathione metabolism. Total glutathione (GSx) increased at low dose CUR (≤ 10 mg.l^−1^–28 µM-) (up to +121% in MCF7 cells, P<0.01, and +138% in MDA-MB-231 cells, P<0.01), but decreased at high dose (≥ 25 mg.l^−1^ −70 µM-) (−49%, in MCF7 cells, P<0.02, and −56% in MDA-MB-231 cells, P<0.025). At high dose, in both cell types, GSx-related metabolites decreased, including homocystein, creatine and taurine (−60 to −80%, all, P<0.05). Together with glutathione-S-transferase actvity, data established that GSx biosynthesis was upregulated at low dose, and GSx consumption activated at high dose. Another major target, in both cell types, was lipid metabolism involving, at high doses, accumulation of polyunsaturated and total free fatty acids (between ×4.5 and ×11, P<0.025), and decrease of glycerophospho-ethanolamine and -choline (about −60%, P<0.025). Multivariate statistical analyses showed a metabolic transition, even a biphasic behavior of some metabolites including GSx, between low and high doses. In addition, CUR at 10 mg.l^−1^ in cotreatment with DTX induced modifications in glutathione metabolism, lipid metabolism, and glucose utilization. Some of these changes were biphasic depending on the duration of exposure to CUR.

**Conclusions/Significance:**

Metabolomics reveals major metabolic targets of CUR in breast cancer cells, and biphasic responses that challenge the widely accepted beneficial effects of the phytochemical.

## Introduction

Curcumin (diferuloylmethane or 1,7-bis-(4-hydroxy-3-methoxyphenol)-1,6-heptadiene-3,5-dione, CUR) is the major active compound present in extracts from the rhizome of *Curcuma longa* (Zingiberaceae). Commercial grade CUR is a mixture containing high proportion -about 80%- pure CUR, with about 20% other curcuminoids demonstrating similar biological activity [1]. Therefore findings about these mixtures are most often attributed to CUR. CUR belongs to the pharmacopoeia of Asian traditional medicine or alternative medicine to treat inflammatory diseases and a wide range of disorders. Given the role of inflammation in the promotion of chronic human diseases including Alzheimer’s disease, chronic obstructive pulmonary disease, cataract, diabetes, and cancer, CUR has deserved extensive research. In the oncology field, CUR was reported to exert anti-proliferative, anti-inflammatory, anti-angiogenic and pro-apoptotic effects in various tumor types *in vitro* [2], [3] and *in vivo* [4], [5]. In addition, CUR used in association with anticancer agents has been shown to protect against cisplatin toxicity [6], and to sensitize tumor cells to gemcitabine [7] or paclitaxel [8].

At the molecular level, CUR has been shown to target the transcription factor NF-κB and gene expression related to NF-κB, including immune response, cell adhesion, differentiation, proliferation, angiogenesis and apoptosis [9], and the transcription factor Nrf2 and gene expression related to Nrf2, including anti-oxidant enzymes (glutamate cysteine ligase, GCL) and phase-2 detoxification proteins [10], [11], [12]. Other targets of CUR include thioredoxin reductase [13], cyclooxygenase-2 (COX-2) and phospholipase A_2_ (PLA_2_) [14].

A growing body of evidence establishes that CUR exhibits not only anti- but pro-oxidant activities, and that anti-inflammatory and anti-proliferative effects are linked to pro-oxidant activities [15], [16]. The anti-oxidant effect, mostly reported at low dose, involves scavenging of reactive oxygen species (ROS) [17], attenuation of lipid peroxidation during oxidative stress [18] and activation of signalling pathways yielding increased expression of heat-shock proteins, anti-oxidant enzymes and anti-apoptotic proteins. In contrast, the less studied pro-oxidant effect, mostly occuring at high dose, involves oxidative DNA damage, protein and membrane lipid peroxidation and apoptosis [15]. The activation of the anti-oxidant response is considered to be at the origin of most beneficial effects of CUR in the prevention and relief of oxidative stress-associated degenerative diseases. In contrast, in the cancer field, the beneficial effects of CUR are challenged by the fact that many drugs kill tumor cells through induction of oxidative stress [19]. Furthermore, it has been reported that CUR can inhibit apoptosis induced by ROS generating chemotherapy [20], [21]. However, most reports on antitumor effects of CUR relate them to anti-inflammatory activities that are generally underpinned by pro-oxidant effects. These appearently paradoxical data may relate to the fact that anti- and pro-oxidant effects of CUR fit the response to a number of cell stressing agents which is biphasic as a function of dose with a stimulating phase followed by a toxic phase. This type of response, called hormesis, is frequently encountered in the field of nutripharmaceutics.

Because there is much expectation on the use of CUR in cancer treatment, there is a need for identifying biomarkers of the response and improving knowledge on the mechanisms of the response. Although metabolomics is increasingly used to evaluate mechanisms of action of various types of anticancer agents [19], [22], [23], it was little applied to tumor cell response to nutritional agents [24], and, to our knowledge, not yet to CUR. In this article, we applied ^1^H-NMR spectroscopy-based metabolomics to investigate the response of MCF7 and MDA-MB-231 breast cancer cells to increasing doses of CUR and that of MCF7 cells to CUR in cotreatment with docetaxel (DTX), a therapeutic combination that has been proposed in advanced and metastatic breast cancer [25]. In both cell types, a major metabolic target of CUR was glutathione (GSx) metabolism. GSx varied biphasically with CUR dose. GSx-related metabolites and glutathione *S-*transferase (GST) activity helped elucidating the mechanisms of this variation. Another major target of both cell types was lipid metabolism. In addition, the combination of DTX to CUR elicited duration of association-dependent metabolic transitions, including a biphasic response of glucose utilization. Hormetic behavior could account for apparently paradoxical responses to CUR, but challenges the widely accepted beneficial effects of the phytochemical.

## Materials and Methods

### Chemicals and Reagents

Commercial grade CUR was obtained from Sabinsa Corporation (Piscataway, USA). It contained approximately 80% pure CUR as confirmed by high performance liquid chromatography tandem mass spectrometry, and two other minor fractions: demethoxycurcumin and bisdemethoxycurcumin. As stated before, the effects of minor fractions closely mimic those of pure CUR. For the sake of simplicity, we thus designated the mixture by CUR in the following. CUR was prepared so as to obtain the final concentration of 0.5, 2.5, 10, 25, and 50 mg.l^−1^ in 0.5% dimethylsulfoxide (DMSO, Merckeurolab, Strasbourg, France), corresponding to 1.4, 7, 28, 70 and 140 µM, respectively. Docetaxel (DTX) was purchased from Sigma Aldrich (Saint Quentin Fallavier, France), dissolved in DMSO and administered so as to obtain a final concentration of 1 nM or 5 µM in 0.5% DMSO in cell culture flasks. RPMI-1640 and Eagle’s MEM-Glutamax media, MEM vitamin solution, sodium pyruvate, non essential amino acids, phosphate buffered saline (PBS) solution and gentamicin base were from Gibco Invitrogen (Cergy Pontoise, France). Heat-inactivated fetal calf serum (FCS) was from Bio West (Nuaillé, France). Low melting point agarose, sodium lauroyl sarcosinate, ethidium bromide solution, Hoechst 33342 and trypan blue dyes were from Sigma-Aldrich (Saint Quentin Fallavier, France). D_2_O for NMR use was from SDS (Peypin, France).

### Cell Culture

Human MCF7 breast carcinoma cells and human MDA-MB-231 breast carcinoma cells were purchased from the European collection of cell cultures (Salisbury, UK). These cell types differ in that MCF7 cells express hormonal (estrogen and progesterone) receptors, while MDA-MB-231 cells do not. Cells were maintained as a monolayer culture at 37°C in humidified atmosphere containing 5% CO_2_ in Eagle’s MEM-Glutamax medium supplemented with 10% FCS, 1% MEM vitamin solution, 1% sodium pyruvate, 1% non essential amino acids and 0.04% gentamicine base. Cells were plated in triplicate into 96-well plates at a density of 3.5×10^4^ cells/well for DNA content measurement, membrane integrity assessment and microscopy analysis, or T180 flasks (10×10^6^ cells/flask) for NMR spectroscopy analysis.

### Cell Treatment

Cells were exposed to CUR at 0.5, 2.5, 10, 25, and 50 mg.l^−1^ for 24 h for the analysis of dose-dependent effect, and at 10 mg.l^−1^ for 24, 48, 72, and 96 h for the analysis of time-dependent response. Measurements in treated MCF7 and MDA-MB-231 cells were compared to control MCF7 and MDA-MB-231 cells (0.5% DMSO, CTL). When following time effects, measurements relative to control values were matched for time. Cotreatments of CUR with DTX consisted in exposure of MCF7 cells for 72 h to DTX at 1 nM (low dose, L) or at 5 µM (high dose, H), in combination with 10 mg.l^−1^ CUR for 24 h (CO-L-24 h and CO-H-24 h, DTX at low and high dose, respectively), 48 h (CO-L-48 h and CO-H-48 h, DTX at low and high dose, respectively), 72 h (CO-L-72 h and CO-H-72 h, DTX at low and high dose, respectively), and 96 h (CO-L-96 h and CO-H-96 h, DTX at low and high dose, respectively). Before analysis, cells were washed twice with PBS (100 µl per well or 20 ml per flask) to remove detached cells.

### DNA Content Measurement

DNA content of MCF7 and MDA-MB-231 breast cancer cells was measured after cell lysis, using Hoescht 33342 staining. DNA content was an index of biomass and ploidy of attached cells. Briefly, 100 µl of a 0.01% (m/v) sodium dodecyl sulfate solution in sterile distilled water was distributed into each well. Cells were then incubated for 1 h at room temperature and frozen at −80°C for 1 h. After thawing (approximately 15 min), 100 µl of Hoechst 33342 solution at 30 µg.ml^−1^ in a hypersaline buffer (10 mM Tris–HCl, pH 7.4, 1 mM EDTA, and 2 M NaCl) was added to each well. Plates were incubated in this solution for 1 h and protected from light at room temperature on a plate shaker. Fluorescence was then measured on a microplate fluorometer (Fluoroskan Ascent FL, Thermolabsystem, Helsinki, Finland) using an excitation wavelength of 360 nm and an emission wavelength of 460 nm.

### Comet Assay

The measured tail DNA value corresponded to the amount of DNA in the comet tail, increased proportionally with the number of DNA strand breaks induced by oxidative stress. DNA strand breaks in MCF7 cells were quantified using the alkaline version of the Comet assay [26]. Ten µl of treated MCF7 cell suspension (approximately 20,000 cells) were mixed with 110 µl of 0.6% low melting point agarose in RPMI-1640 at 37°C. Subsequently, 110 µl of the mixture were layered onto a slide pre-coated with thin layer of 1% agarose, and immediately covered with a cover-glass. Slides were left for 10 min on ice in order to allow the agarose to solidify. After gently removing the cover-glass, the slides were immediately immersed in an ice-cold freshly prepared lysis solution (2.5 M NaCl, 10 mM Na_2_-EDTA, 10 mM Tris hydroxymethyl-aminomethane, 1% sodium lauroyl sarcosinate, 1% Triton 100X and 10% DMSO, pH 10) to lyse the cells and to allow DNA unfolding. After 1 h in the dark at 4°C, the slides were immersed in freshly prepared alkaline electrophoresis buffer (300 mM NaOH, 200 mM Na_2_-EDTA, pH 13) for unwinding (25 min) and then electrophorezed (25 V/300 mA, 25 min). All the steps were carried out under minimal illumination. Once electrophoresis was completed, slides were neutralized (3×5 min, 0.4 M Tris, pH 7.5). The dried microscope slides were stained with 20 mg.l^−1^ ethidium bromide (50 µl per slide) and covered with a cover-glass. Tail DNA was assessed under fluorescence microscopy using the Comet Analysis Software 4.0 (Kinetic Imaging Ltd., Liverpool, UK). Fifty randomly selected cells per slide were analyzed for each triplicate condition.

### Total Glutathione *S-*transferase Activity Assay

Total (cytosolic and microsomal) glutathione *S-*transferase (GST) activity was assayed by conjugation of 1-chloro-2,4-dinitrobenzene (CDNB) with reduced glutathione (GSH), using the Glutathione *S-*Transferase Assay Kit (Cayman Chemical Company, Ann Arbor, MI, USA). MCF7 cells were harvested using a rubber policeman and centrifugated at 1,500×g for 10 min at 4°C. Cell pellets were resuspended in 1 ml of cold buffer (100 mM potassium phosphate, pH 7.0, containing 2 mM EDTA). Cell suspensions were then sonicated for 30 s on ice and centrifugated at 10,000×g for 15 min at 4°C. Supernatant was used for GST measurement. Ten µl of CDNB, 20 µl of GSH, and 150 µl of assay buffer (100 mM potassium phosphate, pH 6.5, containing 0.1% Triton X-100) were added to 20 µl of sample. Non enzymatic sample contained 170 µl of assay buffer and 20 µl of GSH. The formation of the *S-*conjugate induced an increase in absorbance at 340 nm monitored every 60 s for 6 min, using a DU800 spectrophotometer (Beckman Coulter, Villepinte, France) at 25°C.

### ^1^H-NMR Spectroscopy-based Metabolomics

Attached MCF7 and MDA-MB-231 breast cancer cells were collected by centrifugation (1,500×g for 10 min at 4°C). Cell pellets were washed twice with 1 ml D_2_O containing 1% PBS and frozen at –80°C until analysis. Five to 10×10^6^ intact cells were used for each NMR spectroscopy acquisition. NMR Spectroscopy was performed on a small bore 500 MHz Avance DRX spectrometer (Bruker Biospin, Karlsruhe, Germany) equipped with a high resolution magic angle spinning (HRMAS) probe. Unprocessed cell pellets were set into 4 mm-diameter 50 µl free volume zirconium oxide rotor tubes. Rotors were spun at 4 kHz, and cooled at 4°C using the BCU-05 temperature unit. One-dimensional (1D) proton NMR spectra were obtained using a Nuclear Overhauser Enhancement spectroscopy sequence with low power water signal presaturation (NOESYPR) during both the 3.8-s relaxation delay and the 100-ms mixing time of the sequence. The spectral width was 12 ppm with 16K complex data points and 32 transients. This resulted in 2∶50 min acquisition duration. After Fourier transformation, low order phase correction and baseline spline correction were applied in a standardized way. Signal attribution in spectra was done as previously described [27], [28]. Quantification of one-dimensional (1D) NMR spectra was performed using the Topspin 2.0 software (Bruker Biospin, Karlsruhe, Germany). A two-dimensional (2D) NMR spectrum was recorded immediately after the 1D NMR spectrum, using a Total Correlation Spectroscopy (TOCSY) sequence involving water signal suppression at low power, 6-ppm spectral bandwidth along both frequency axes, 256 samples along the first axis, and 2K samples along the second axis, 75-ms mixing time during which was applied the spin-lock pulse train (DIPSI-2), 1-s relaxation delay, and 16 repetitions. The 2D NMR spectrum duration was 1∶41 h. TOCSY spectra were reconstructed at both high (2K×256) and moderately lower (256×256) spectral resolution for signal attribution and quantification, respectively. Baseline correction was applied using a second order polynomial. Then spectra were transferred to an Excel worksheet (Microsoft Co.), and processed using a homebuilt routine designed to automatically compute cross-peak volumes of selected spectrum signals. Retained metabolites are listed in Table 1. From quantified 1D and 2D NMR signals, relative metabolite concentrations were determined according to the method of standardization to protein signals [22], [28].

**Table 1:** Proton chemical shift of signals used for metabolite quantification.

Chemical shift	NMR technique	Metabolite	Abbreviation
*Methionine and glutathione metabolism derivatives*			
(1.72,2.13)×3.13	2D	Polyamines	Ply
2.17×2.72	2D	Homocysteine	Hcy
3.93;3.03	1D	Total creatine	tCr
(2.55,4.56)×2.17	2D	Total glutathione	GSx
(2.41,2.51)×4.17	2D	Oxoproline	OP
2.63×3.35	2D	Hypotaurine	hTa
3.27×3.43	2D	Taurine	Tau
*Lipid and phospholipid metabolism derivatives*			
1.92	1D	Acetate	Ace
0.88×1.33	2D	Total fatty acids	tFA
2.79×5.33	2D	Polyunsaturated fatty acids	PUF
2.55×2.75	2D	Citrate	Cit
3.22×3.99	2D	Phosphoethanolamine	PE
3.30×4.12	2D	Glycerophosphoethanolamine	GPE
3.55×4.07	2D	Choline	Cho
3.62×4.18	2D	Phosphocholine	PC
3.26	1D	Phosphatidylcholine	PtC
3.66×4.42	2D	Cytidine diphosphocholine	CDPC
3.66×4.34	2D	Glycerophosphocholine	GPC
*Bioenergetic metabolism derivatives*			
8.53	1D	Adenosine tri-phosphate	ATP
8.35	1D	Adenosine mono-phosphate	AMP
7.98	1D	Uridine diphosphate conjugates	UDPX
1.34×4.11	2D	Lactate	Lac
(3.54,3.61)×3.28	2D	Myo-inositol	MyI
(3.76,4.13)×4.02	2D	Gluconate	Gna
*Glutamate metabolism derivatives*			
2.06×3.76	2D	Glutamate	Glu
2.12×2.46	2D	Glutamine	Gln
2.03×4.15	2D	Proline	Pro
1.47×3.77	2D	Alanine	Ala
(2.88,2.95)×3.99	2D	Asparagine	Asn
(2.70,2.80)×3.89	2D	Aspartate	Asp
(2.50,2.70)×4.40	2D	N-acetyl-aspartate	NAA
(1.68,1.92)×3.23	2D	Arginine	Arg
*One-carbon metabolism derivatives and others*			
3.56	1D	Glycine	Gly
8.45	1D	Formate	For
1.32×3.58	2D	Threonine	Thr
1.90×3.77	2D	Lysine	Lys
3.13×3.99	2D	Phenylalanine	Phe

Single chemical shift values or 2 chemical shifts separated by a semi-colon indicate 1D HRMAS ^1^H-NMR spectrum measurement; two chemical shift values separated by a multiplicative sign indicate a measured cross-peak in 2D HRMAS ^1^H-NMR spectra; two chemical shift values within parentheses separated by a multiplicative sign from another chemical shift indicate 2 measured cross-peaks in 2D HRMAS ^1^H-NMR spectra.

### Data Processing and Statistical Analysis

Comparisons between groups were performed using the non parametric Mann-Whitney test. Differences were considered statistically significant for P<0.05. Principal component analysis (PCA) using the metric of Spearman was applied to the different data sets (dose-related data, time-related data, and cotreatment data). Metabolic interpretation of principal components was done using loading plots of axes. Partial least squares regression-discriminant analysis (PLS-DA) was applied to reveal metabolites associated with common response to CUR in both MCF7 and MDA-MB-231 cell types, and to check for metabolites displaying a hormetic behavior. In the latter case, the tested model consisted in a positive phase between CTL and low dose samples (assigned class values of 0 and +1, respectively), then a negative phase (assigned class values of 0 and −1, at 25 and 50 mg.l^−1^, respectively). The variable importance in the projection (VIP) was calculated for each metabolite. Criteria of quality of modeling were R2X, the cumulative modeled variation in metabolite data, R2Y, the cumulative modeled variation in class values, and Q2, the cumulative predicted variation in metabolite data according to cross-validation. These parameters range between 0 and 1, where 1 indicates a perfect fit. The highest values of VIP identified metabolites that complied best with the tested model. Multivariate statistical analyses were performed using the XLSTAT 7.5 software (Addinsoft, Paris, France).

## Results

### Dose-dependent Response of Breast Cancer Cells to CUR Reveals Metabolic Targets

#### Cellular and biochemical measurements

**Figure 1:**
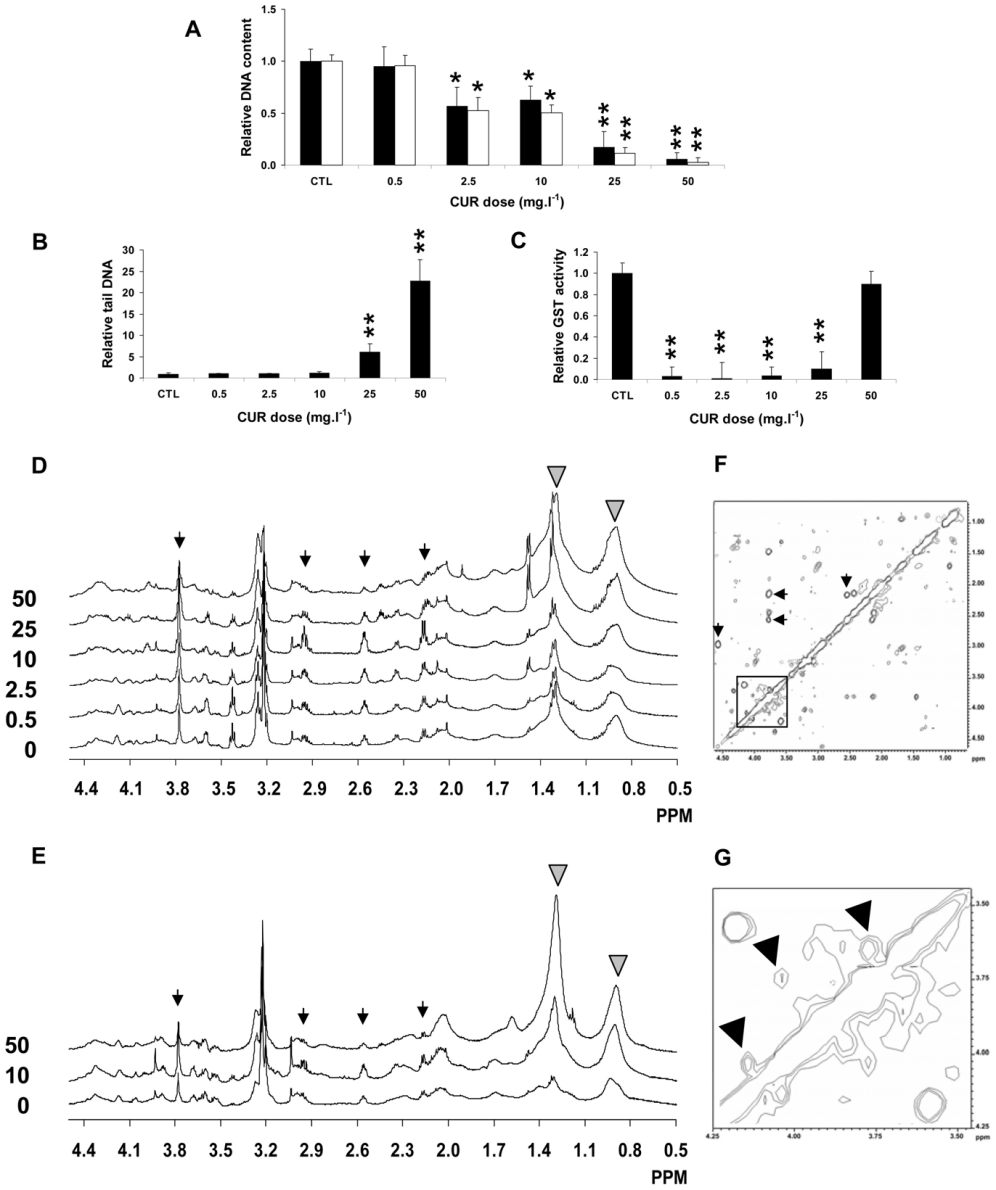
Dose-dependent response to CUR. A- Cellular DNA content in attached MCF7 (black bars) and MDA-MB-231 (white bars) breast cancer cells exposed to increasing concentrations (0.5, 2.5, 10, 25, and 50 mg.l^−1^) of CUR for 24 h, measured using the Hoechst fluorescence intensity assay. *, P<0.05, **, P<0.01, CUR *vs.* CTL, Mann-Whitney test. B- DNA fragmentation expressed in tail DNA in MCF7 tumor cells exposed to increasing concentrations (0.5, 2.5, 10, 25, and 50 mg.l^−1^) of CUR for 24 h, measured using the alkaline Comet assay. *, P<0.05, CUR *vs.* CTL, Mann-Whitney test. C- Total glutathione *S-*transferase (GST) activity in MCF7 tumor cells exposed to increasing concentrations (0.5, 2.5, 10, 25, and 50 mg.l^−1^) of CUR for 24 h, measured using the Cayman Chemical Company assay kit. **, P<0.01, CUR *vs.* CTL, Mann-Whitney test. D- Set of typical 1D HRMAS ^1^H-NMR spectra of MCF7 breast cancer cells in the 0.5–4.5 ppm spectral range. From bottom to top, cells exposed to 0, 0.5, 2.5, 10, 25, and 50 mg.l^−1^ CUR. Thin arrows, signals from glutathione (GSx) at positions 2.25, 2.55, 2.98, and 3.78 ppm. Grey arrowheads, signal from total free fatty acids (tFA) at positions 1.30 and 0.89 ppm. E- Set of typical 1D HRMAS ^1^H-NMR spectra of MDA-MB-231 breast cancer cells. From bottom to top, cells exposed to 0, 10, and 50 mg.l^−1^ CUR. Arrows, as in Fig. 1D. F- Two-dimensional HRMAS ^1^H-NMR spectra of intact MCF7 tumor cells, here from the 25 mg.l^−1^ CUR group. Thin arrows, cross-peaks of glutathione (GSx) at positions 2.25×2.55, 2.25×3.78, 2.55×3.78, and 2.98×4.55 ppm×ppm. Square, area containing gluconate (Gna) signals. G- Square area magnified from the spectrum in Fig. 1F. Arrowheads, cross-peaks from Gna at positions 4.13×4.02, 4.02×3.76, and 3.76×3.66 ppm×ppm. Gna was quantified from the first 2 cross-peaks.

Relative DNA content decreased in response to CUR at 2.5 mg.l^−1^ (×0.57±0.18 and ×0.52±0.13, MCF7 and MDA-MB-231, P<0.05), 10 mg.l^−1^ (×0.63±0.13 and ×0.50±0.08, MCF7 and MDA-MB-231,P<0.05), then even more at 25 mg.l^−1^ (×0.18±0.15 and ×0.12±0.05, MCF7 and MDA-MB-231, P<0.01) and 50 mg.l^−1^ (×0.06±0.06 and ×0.03±0.04, MCF7 and MDA-MB-231, P<0.01) (Fig. 1A). DNA oxidative damage, expressed by tail DNA, increased in attached MCF7 cells in response to CUR at 25 mg.l^−1^ (×6.2±1.9, P<0.01) and 50 mg.l^−1^ (×23±5, P<0.01) (Fig. 1B). GST activity dramatically dropped in cells treated at 0.5 mg.l^−1^ (×0.03±0.09, P<0.001), 2.5 mg.l^−1^ (×0.01±0.15, P<0.001), 10 mg.l^−1^ (×0.04±0.08, P<0.001), and 25 mg.l^−1^ (×0.10±0.16, P<0.001), whereas it resumed to CTL levels at 50 mg.l^−1^ (×0.90±0.12, P = NS) (Fig. 1C).

#### Metabolite profiling with increasing doses of CUR

Dose-related 1D HRMAS ^1^H-NMR spectra showed prominent biphasic alterations of GSx. GSx accumulated at low doses (0.5 to 10 mg.l^−1^) whereas it decreased at high doses (25 and 50 mg.l^−1^), in both MCF7 (Fig. 1D) and MDA-MB-231 (Fig. 1E) cells. Also free fatty acids accumulated at high dose only, in both MCF7 and MDA-MB-231 cells.

**Figure 2:**
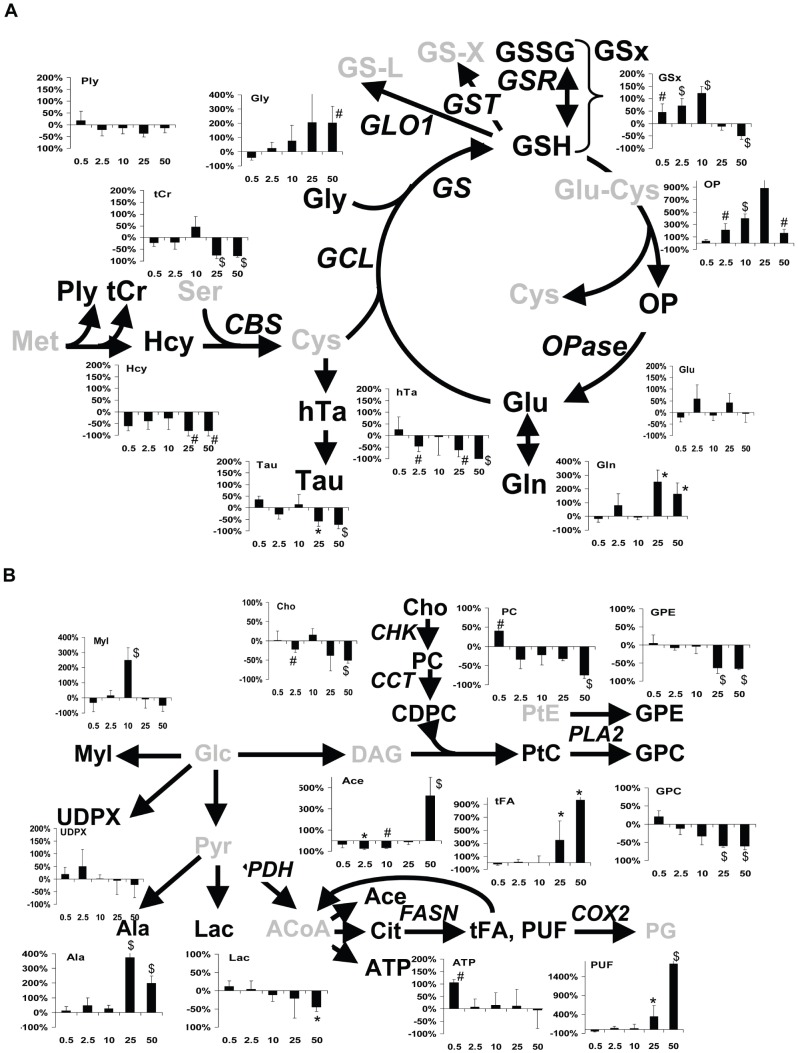
Co-mapping of dose-related metabolite changes with metabolic schemes. A- Glutathione cycle and metabolism. Abbreviations: GS-X, conjugated GSH; GSSG, oxidized GSH; GS-L, lactoyl-glutathione; Glu-Cys, glutamylcysteine; Cys, cysteine; Met, methionine; Ser, serine; GST, glutathione *S-*transferase; GSR, glutathione *S-*reductase; GLO1, glyoxalase-1; GS, glutathione synthetase; GCL, glutamate-cysteine ligase; OPase, oxoprolinase, CBS, cystathionine beta-synthase. Other metabolite abbreviations, as in Table 1. Italicized, enzymes; Grey, undetected/unmeasured metabolites; #, P<0.05; *, P<0.025; $, P<0.01, Mann-Whitney test. B- Glycolysis and lipid metabolism. Abbreviations: PtE, phosphatidylethanolamine; Glc, glucose; DAG, diacylglycerol; Pyr, pyruvate; ACoA, acetyl-CoA; PG, prostaglandin; CHK, choline kinase; CCT, choline cytidylyltransferase; PDH, pyruvate dehydrogenase; PLA_2_, phospholipase A_2_; FASN, fatty acid synthase; COX-2, cyclooxygenase-2. Other metabolite abbreviations, as in Table 1. #, P<0.05; *, P<0.025; $, P<0.01, Mann-Whitney test.

Assignment of signals in 2D HRMAS ^1^H-NMR spectra revealed an unusual metabolite, only visible in 2D NMR spectra of CUR-treated cells, corresponding to gluconate (Gna) (Fig. 1F,G). A total of 37 metabolites were quantified (Table 1). In MCF7 cells, among glutathione metabolism derivatives, GSx increased continuously until 10 mg.l^−1^ (+45% at 0.5 mg.l^−1^, P<0.05; +72% at 2.5 mg.l^−1^, P<0.01; +121% at 10 mg.l^−1^, P<0.01), oxoproline (OP) accumulated at 2.5 and 10 mg.l^−1^ (×3, P<0.05, and ×5, P<0.025, respectively), and hypotaurine (hTa) decreased transiently to −47% (P<0.05) (Table 2, and Fig. 2). Among glucose metabolization derivatives, MyI reached a peak level at 10 mg.l^−1^ (×3.5, P<0.01), and among lipid metabolism derivatives, Ace decreased by -70% (P<0.025), and PE first decreased then reached a ×2.2 peak level (P<0.05). No significant changes were found in tFA and PUF levels at low dose. In contrast, at high dose (25 and 50 mg.l^−1^), tFA and PUF dramatically increased (×4.5, P<0.025 and ×11, P<0.025, and ×4.5, P<0.025 and ×18, P<0.01, respectively), two phospholipid derivatives, GPE and GPC, decreased (about -60%, P<0.025), and GSx decreased (−11% at 25 mg.l^−1^ and −49% at 50 mg.l^−1^, P<0.01). Several glutathione-related derivatives decreased (Hcy, −80% and −81%, P<0.05; tCr, −74% and −78%, P<0.01; Tau, −57% and −73%, P<0.025; and hTa, −63% and −100%, P<0.05, at 25 and 50 mg.l^−1^ respectively) (Table 2 and Fig. 2).

**Table 2:** CUR dose-related data in MCF7 cells (fold variation *vs.* 24 h controls).

CUR dose (mg.l^−1^)													
	0		0.5		2.5		10		25		50		
	Mean	SD	Mean	SD	Mean	SD	Mean	SD	Mean	SD	Mean	SD	
n	7		3		3		3		3			3	
tFA	1	0.66	0.8	0.71	1.12	0.15	1	0.08	4.47	2.23*	10.62	7.10*	
PUF	1	1.61	0.52	0.63	1.41	0.38	1.32	0.98	4.45	1.46*	18.37	11.59$	
Lac	1	0.25	1.11	0.18	1.04	0.29	0.89	0.23	0.79	0.66	0.55	0.16*	
Ace	1	0.63	0.65	0.38	0.25	0.11*	0.32	0.07#	0.89	0.33	5.26	2.12$	
NAA	1	0.32	0.96	0.58	0.9	0.07	0.91	0.05	0.75	0.12	0.41	0.29*	
MyI	1	0.61	0.68	0.7	1.14	0.42	3.49	0.99$	0.92	0.73	0.5	0.49	
Glu	1	0.24	0.79	0.25	1.59	0.73	0.87	0.28	1.41	0.49	0.95	0.47	
Gln	1	0.46	0.84	0.33	1.8	1.05	0.94	0.22	3.51	1.07*	2.62	1.01*	
Pro	1	0.37	0.95	0.37	0.97	0.4	1.21	0.35	1.3	0.22	0.54	0.08#	
Asn	1	0.31	0.9	0.25	0.7	0.2	0.87	0.32	0.83	0.4	0.91	0.05	
Asp	1	0.66	1.03	1.19	0.95	0.81	0.17	0.13#	0.74	0.55	0.24	0.34	
Arg	1	0.33	0.91	0.11	0.7	0.12	0.82	0.17	1.29	0.17	0.75	0.05	
Ply	1	0.41	1.18	0.48	0.79	0.32	0.87	0.31	0.64	0.18	0.86	0.25	
Hcy	1	0.69	0.39	0.24	0.61	0.44	0.73	0.6	0.2	0.28#	0.19	0.26#	
tCr	1	0.31	0.78	0.19	0.8	0.36	1.44	0.55	0.26	0.18$	0.22	0.07$	
hTa	1	0.44	1.26	0.66	0.53	0.28#	0.93	0.96	0.37	0.37#	0	0.00$	
Tau	1	0.29	1.35	0.18	0.71	0.24	1.14	0.54	0.43	0.28*	0.27	0.22$	
GSx	1	0.18	1.45	0.42#	1.72	0.35$	2.21	0.35$	0.89	0.19	0.51	0.16$	
Gly	1	0.71	0.58	0.2	1.25	0.51	1.75	1.34	3.06	3.59	3.03	1.42#	
Ala	1	0.42	1.12	0.33	1.47	0.64	1.26	0.28	4.74	1.10$	2.98	0.63$	
Lys	1	0.46	1.2	0.10#	1.06	0.4	1.17	0.54	1.87	0.42#	1.63	0.68#	
Phe	1	0.22	0.94	0.28	0.84	0.13	0.71	0.11*	1.4	0.07#	0.91	0.08	
Thr	1	0.27	1.14	0.35	0.79	0.53	0.91	0.41	1.62	0.50#	1.03	0.28	
PE	1	0.69	0.56	0.08#	0.93	0.64	2.15	0.91#	0.69	0.3	0.94	0.28	
GPE	1	0.14	1.05	0.28	0.92	0.08	0.96	0.25	0.37	0.19$	0.35	0.03$	
Cho	1	0.13	1.01	0.29	0.78	0.10#	1.16	0.19	0.62	0.5	0.49	0.10$	
PC	1	0.41	1.4	0.09#	0.66	0.31	0.78	0.32	0.68	0.06	0.25	0.12$	
CDPC	1	0.23	1.07	0.02	1.13	0.43	1.25	0.28	0.89	0.3	1.07	0.61	
PtC	1	0.21	1.19	0.27	1	0.43	1.14	0.47	0.62	0.3	1.17	0.49	
GPC	1	0.34	1.21	0.2	0.88	0.21	0.67	0.29	0.4	0.04$	0.39	0.11$	
Gna	1	0.79	0.78	0.1	0.92	0.7	1	0.3	3.28	1.85#	1.44	0.89	
Cit	1	0.44	0.92	0.11	0.47	0.33	1.21	1.02	0.71	0.1	1.23	1.04	
OP	1	0.93	1.32	0.33	3.11	1.26#	4.98	0.88$	9.83	8.8	2.61	0.80#	
ATP	1	0.57	2.05	0.16#	1.07	0.39	1.14	0.61	1.11	0.82	0.95	0.91	
For	1	0.52	1.19	0.04	0.74	0.26	0.78	0.45	0.29	0.24#	0.32	0.14$	
AMP	1	0.46	0.16	0.03#	0.77	0.48	0.66	0.59	0.14	0.09$	0.24	0.13#	
UDPX	1	0.22	1.19	0.34	1.5	0.82	1	0.19	0.94	0.67	0.78	0.63	

Mean control value is set to 1 while dispersion of control data is maintained. Metabolite abbreviations, see Table 1. SD, standard deviation. #, P<0.05; *, P<0.025; $, P<0.01, Mann-Whitney test.

Mean control value is set to 1 while dispersion of control data is maintained. Metabolite abbreviations, see Table 1. SD, standard deviation. *, P < 0.025; $, P < 0.01, Mann-Whitney test.

In MDA-MB-231 cells at low dose (10 mg.l^−1^) CUR, similar findings were obtained with GSx (+138%, P<0.01), no significant changes in tFA and PUF, and a high level of MyI (+241%, P<0.025). At high dose (50 mg.l^−1^) CUR, like in MCF7 cells, GSx decreased (-56%, P<0.025), with evidence of exhausted transsulfuration (Hcy, tCr and Tau all decreased, P<0.05), free fatty acid accumulation (tFA: ×7.8, P<0.01 and PUF: ×9.5, P<0.025), and GPE and GPC decreased (both, P<0.01) (Table S1).

**Figure 3:**
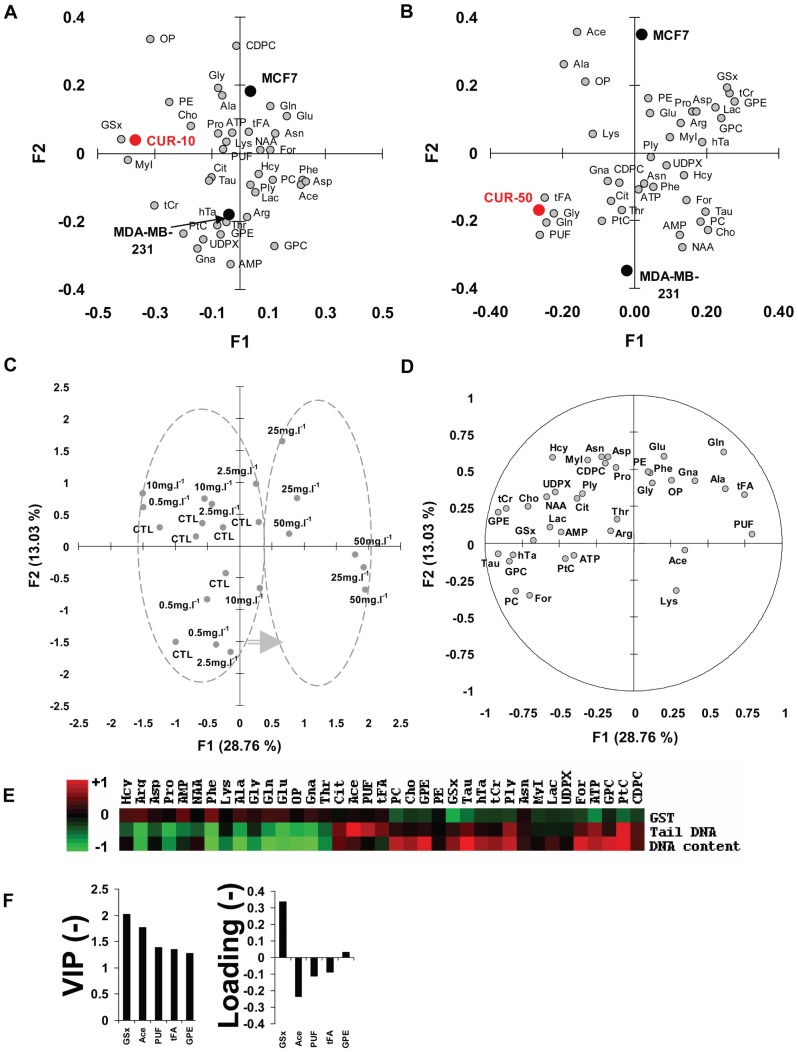
Multivariate analysis of CUR dose-related metabolite data. A- PLS-DA of metabolite data obtained in MCF7 and MDA-MB-231 cells types responding to 10 mg.l^−1^ CUR. Comapping of metabolites and 3 factors (cell type: MCF7 and MDA-MB-231) and common response to CUR at 10 mg.l^−1^ (CUR10). Metabolite abbreviations, as in Table 1. B- PLS-DA of metabolite data obtained in MCF7 and MDA-MB-231 cells types responding to 50 mg.l^−1^ CUR. Comapping of metabolites and 3 factors (cell type: MCF7 and MDA-MB-231) and common response to CUR at 50 mg.l^−1^ (CUR50). Metabolite abbreviations, as in Table 1. C- PCA of CUR dose related data. Each score corresponds to an individual measurement and is labeled CTL or the corresponding CUR dose level. F1 and F2, first 2 principal components with the percentage of total variance of data they account for. Two elliptic clouds separate, to the left, the set of CTL and low dose CUR (0.5, 2.5, and 10 mg.l^−1^) samples, and, to the right, the set of high dose CUR (25 and 50 mg.l^−1^) samples. Grey arrow, trajectory between low and high dose clouds. D- Loading plot of PCA. Metabolite abbreviations, as in Table 1. E- Partial correlation of CUR dose-related data with available covariables (DNA content, tail DNA and GST activity). Red, positive correlation; green, negative correlation; left, color scale of correlations, between -1 and +1. F- PLS-DA of metabolite data, after fitting to a hormetic model, showing the VIP plot of the 5 most discriminant metabolites with hormetic behaviour (left). Corresponding loading plot (right). Metabolite abbreviations, as in Table 1.

PLS-DA of metabolite data obtained in MCF7 and MDA-MB-231 cells types responding to 10 mg.l^−1^ CUR showed that the common response to CUR at 10 mg.l^−1^ of both cell types was correlated with high levels of GSx and MyI (Fig. 3A). Similar plot with CUR at 50 mg.l^−1^ revealed that the common response of both cell types to CUR at that dose was correlated with high levels of tFA, PUF, Gln, Gly (Fig. 3B). Together with univariate data (Table 2 and Table S1), these PLS-DA analyses confirm that major targets of CUR at low and high dose include glutathione and lipid metabolism.

#### PCA of dose-related metabolite data

PCA of the whole set of CTL and dose-related data in MCF7 cells revealed two separated clouds along the first axis F1 representing about 29% of the variance of data. The first cloud, to the left, included CTL and low dose (0.5, 2.5, and 10 mg.l^−1^) samples, and the second one, to the right, included high dose (25 and 50 mg.l^−1^) samples (Fig. 3C). The CTL group could not be separated from low dose samples, even using the second factor F2. PCA demonstrated a metabolic transition between low and high doses. The loading plot is given in Fig. 3D. F1 was mostly explained by PUF and tFA which correlated positively with the axis, and Tau, GPE, tCr, GPC which correlated negatively.

#### Partial correlation of metabolites with covariates

The analysis of available covariates in MCF7 cells (DNA content, tail DNA and GST activity) revealed negative correlation between GSx level and GST activity (r = -0.57, P<0.05), positive correlation between Tau and DNA content (r = +0.63, P<0.05), positive correlation between GPC and GPE and DNA content (r = +0.74 and +0.79, P<0.01, respectively), negative correlation between GPC and GPE and tail DNA (-0.58 and -0.70, P<0.05), and positive correlation between tFA, PUF and Ace and tail DNA measurement (r = +0.78, +0.73, and +0.83, P<0.01, respectively) (Fig. 3E).

#### PLS-DA modeling of hormetic-type response

PCA revealed a metabolic transition between low and high doses. GSx level as a function of dose was typically hormetic as shown in Fig. 2A. Therefore, to investigate latent hormetic behavior, MCF7 metabolite data were fitted a biphasic model mimicking GSx response, then processed using PLS-DA. Modeling was obtained with 3 latent factors. The quality of fit was good with R2X = 0.46, R2Y = 0.90 and Q2 = 0.59. Analysis revealed that the first 5 metabolites with the highest VIP, thus exhibiting a hormetic behavior, were GSx, Ace, PUF, tFA and GPE. GSx and GPE increased during the first phase then decreased, and Ace, PUF and tFA decreased during the first phase then increased (Fig. 3F).

### Duration of Exposure-dependent Response of MCF7 Cells to CUR

#### Cellular and biochemical measurements

**Figure 4:**
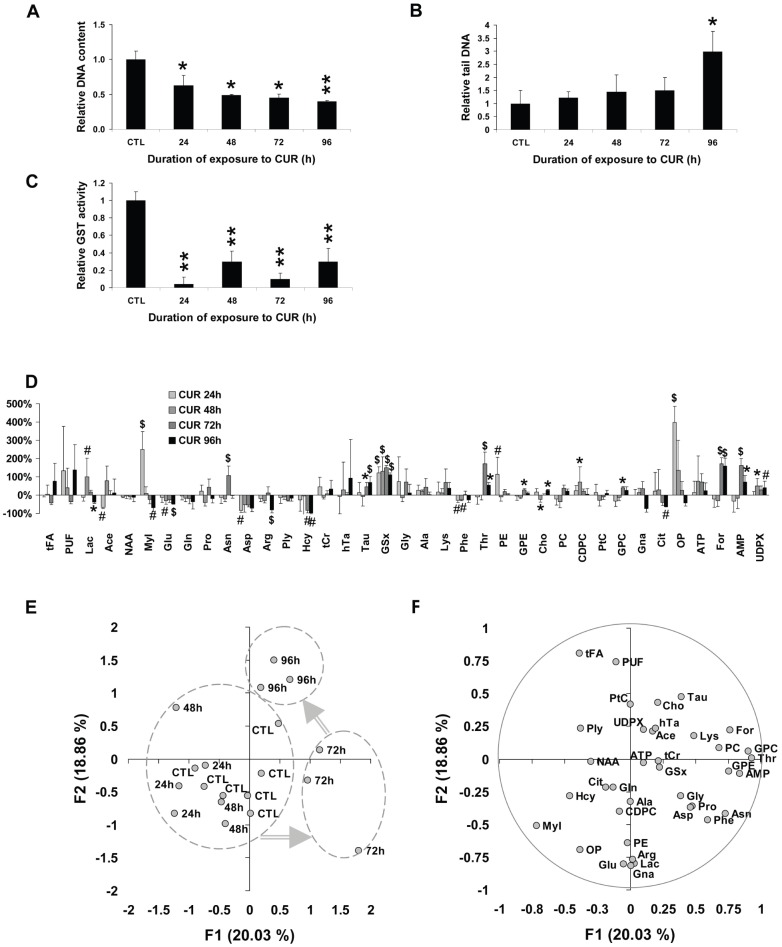
Duration of exposure-related response to CUR. Cells were treated with 10 mg.l^−1^ CUR and followed for 96 h. A- Cellular DNA content in attached MCF7 tumor cells relative to time-matched CTL. DNA content was measured using the Hoechst fluorescence intensity assay. *, P<0.05, **, P<0.01 CUR vs. CTL, Mann-Whitney test. B- Tail DNA relative to time-matched CTL. DNA. Tail DNA was assessed using the alkaline Comet assay. *, P<0.05, CUR vs. CTL, Mann-Whitney test. C- Total GST activity relative to time-matched CTL. GST activity was measured using the Cayman Chemical Company assay kit. **, P<0.01, CUR vs. CTL, Mann-Whitney test. D- Metabolite variations relative to time-matched CTL. #, P<0.05; *, P<0.025; $, P<0.01, CUR *vs.* CTL, Mann-Whitney test. E- PCA of duration of exposure-related data. The scores plot is displayed with individual scores labeled by their duration of exposure, between 24 h and 96 h, with CTL data also displayed. F1 and F2, first 2 principal components with the percentage of total variance of data they account for. Grey arrows, duration of exposure-related trajectory. F- Corresponding loading plot of PCA of duration of exposure-related data. Metabolite abbreviations, as in Table 1.

Relative DNA content decreased with duration of exposure to 10 mg.l^−1^ CUR (from ×0.63±0.09 to ×0.40±0.01, all P<0.05) (Fig. 4A). DNA fragmentation evaluated from tail DNA increased in attached MCF7 cells in response to exposure to CUR for 96 h (×2.98±0.77, P<0.05, Fig. 4B). GST activity was decreased for all durations of exposure (down to ×0.04±0.08 the CTL value, P<0.01, Fig. 4C).

#### Metabolite profiling of duration of exposure-related data

GSx level was high at each time (+121% at 24 h and +112% at 96 h, all P<0.01) (Fig. 4D). OP, a glutathione derivative, was high at 24 h (×5, P<0.01). GSx-related metabolites involved decrease of Hcy (−84 and −97% at 72 h and 96 h, the latter P<0.01) and decrease of Glu derivatives (Glu: −30% at 48 h and −47% at 96 h, P<0.05; Asp: −83% at 24 h, P<0.05; Arg: −80% at 96 h, P<0.01). Meanwhile, there was an increase of Tau (+46% at 72 h and +69% at 96 h, P<0.025). Among lipid metabolism derivatives, Ace was low at 24 h only (−68%, P<0.05) and Cit at 96 h (−62%, P<0.025). tFA and PUF did not vary significantly. A peak level of PE was observed at 24 h (×2.2, P<0.025). Among bioenergetics derivatives, Lac reached a peak level at 48 h (×2, P<0.05) but decreased at 96 h (−39%, P<0.025), and MyI was sequentially high at 24 h and low at 96 h (+250% and −68%, P<0.05, respectively).

#### PCA of duration of treatment-related metabolite data

PCA was performed on duration of treatment-related data (Fig. 4E). A metabolic transition appeared between short duration of exposure (24 h and 48 h) samples and 72 h samples, mostly undelain by axis F1. Another metabolic transition took place between 72 h and 96 h, mostly underlain by axis F2. Axis F1 was explained by GPC, Thr, AMP, For and GPE which correlated positively with the axis, and MyI which correlated negatively (Fig. 4F). Axis F2 was explained by PUF and tFA which correlated positively with the axis and Gna, Glu and Lac which correlated negatively.

### Response of MCF7 Cells to CUR in Cotreatment with Low and High dose DTX

#### Cellular and biochemical measurements

**Figure 5:**
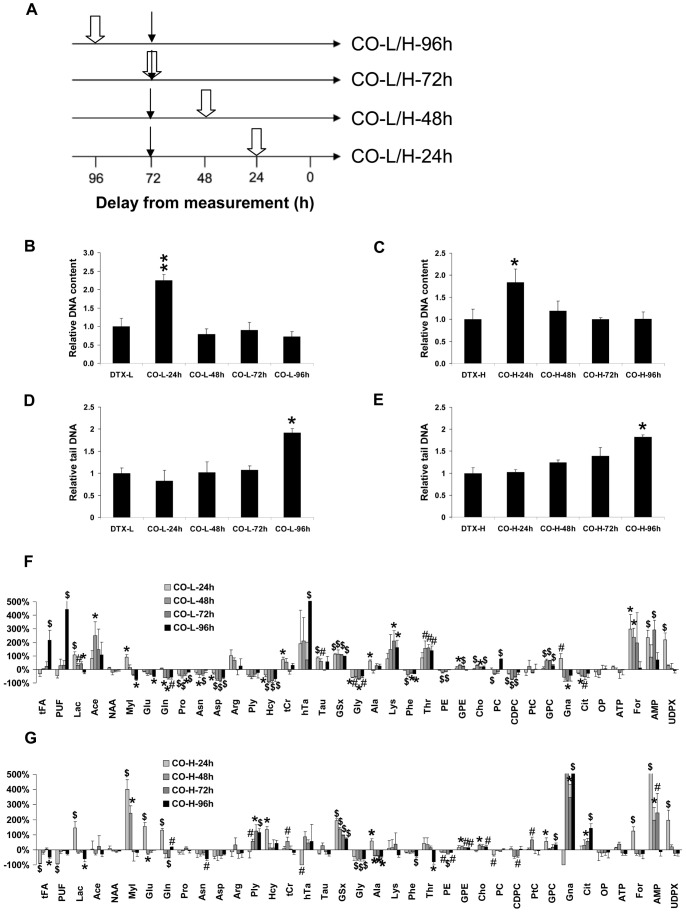
Response of MCF7 cells to co-treatment with CUR and DTX. A- Protocol of cotreatment. Thin arrows, onset of DTX treatment at either low or high dose. White arrow, onset of CUR treatment (10 mg.l^−1^). Right, abbreviations designating each condition: CO, cotreatment; L or H, low dose DTX or high dose DTX; 24–96 h, duration of exposure to CUR at the time of measurement. B- Cellular DNA content in attached MCF7 tumor cells relative to time-matched CTL in combinations with low dose DTX (DTX-L). DNA content was measured using the Hoechst fluorescence intensity assay. C- Cellular DNA content in attached MCF7 tumor cells relative to time-matched CTL in combinations with high dose DTX (DTX-H). D- Tail DNA for cotreatment with low dose DTX (DTX-L) relative to DTX-L. Tail DNA was assessed using the alkaline Comet assay. E- Tail DNA for cotreatment with high dose DTX (DTX-H) relative to DTX-H. F- Metabolite variations as a function of duration of cotreatment with low dose DTX (DTX-L). Abbreviations, as in Table 1. #, P<0.05; *, P<0.025; $, P<0.01, cotreatment vs. DTX-L, Mann-Whitney test. G- Metabolite variations as a function of duration of cotreatment with high dose DTX (DTX-H). Abbreviations, as in Table 1. #, P<0.05; *, P<0.025; $, P<0.01, cotreatment vs. DTX-H, Mann-Whitney test.

The protocol of cotreatment is shown in Fig. 5A. DNA content was decreased with DTX-L and DTX-H at 72 h vs CTL at 72 h (×0.31±0.07, and ×0.15±0.03 the CTL level, both P<0.01). In contrast, tail DNA was poorly altered with DTX-L and DTX-H (×1.33±0.16, and ×0.87±0.20 the CTL level, both P = NS). Measurements were further normalized to 72 h-DTX values. Relative DNA content relative showed a marked increase when duration of exposure to CUR was 24 h with DTX-L (×2.26±0.15 vs ×1±0.22, P<0.01, 24 h cotreatment vs DTX-L alone), also an increase with DTX-H (×1.84±0.30 vs ×1±0.24, P<0.05, 24 h cotreatment vs DTX-H alone). With other durations of cotreatment, relative DNA content was similar to that of the corresponding DTX value (P = NS) (Fig. 5A,B). Tail DNA varied with duration of cotreatment similarly as it did with CUR alone. At 96 h cotreatment with DTX-L, it was ×1.92±0.09 vs ×1.00±0.12, cotreatment vs DTX-L, P<0.05 (Fig. 5D). At 96 h cotreatment DTX-H, it was ×1.83±0.04 *vs.* ×1.00±0.12, cotreatment vs DTX-H, P<0.05 (Fig. 5E).

#### Metabolite profiling in co-treatment

At low dose DTX in cotreatment (Fig. 5F), GSx content was increased by twice whatever the duration of exposure to CUR (from ×2.1 at 24 h to ×2.0 at 96 h, all P<0.01). Other derivatives of GSx metabolism included decreased Hcy (between −39% and −90%, both P<0.025), decreased Gly (−50 to −70%, from 24 h to 96 h, all P<0.05), and increased tCr and Tau at 24 h and/or 48 h (P<0.05). Among lipid metabolism derivatives, tFA and PUF increased at 96 h cotreatment (×3.2, and ×5.4, P<0.01, respectively), Ace increased at 48 h (×3.5, P<0.025), GPC and GPE both increased at 48 h and 72 h (up to +66% and +30%, respectively, P<0.025). Among bioenergetics-related metabolites, Lac increased from 24 to 72 h cotreatment (+107%, +30%, +43%, all P<0.05), and MyI first increased at 24 h (+90%, P<0.025) then decreased at 96 h (−76%, P<0.025).

At high dose DTX in cotreatment (Fig. 5G), GSx increased (from +195% at 24 h to +74% at 96 h, all P<0.01). Hcy increased at 24 h cotreatment (+135%, P<0.025) then returned to the control level. Gly decreased from 48 h to 96 h (about −60%, all P<0.01), tCr increased at 48 h, and hTa decreased at 48 h (both, P<0.05). Among lipid metabolism derivatives, tFA and PUF were low at 24 h and 96 h cotreatment (−90% and −92% at 24 h, P<0.01 and −50% at 96 h for tFA, P<0.025). Lac increased at 24 h (+146%, P<0.01) then decreased at 96 h (−60%, P<0.025). MyI increased at 24 and 48 h (up to ×5, P<0.025).

#### PCA of cotreatment-related metabolite data

**Figure 6:**
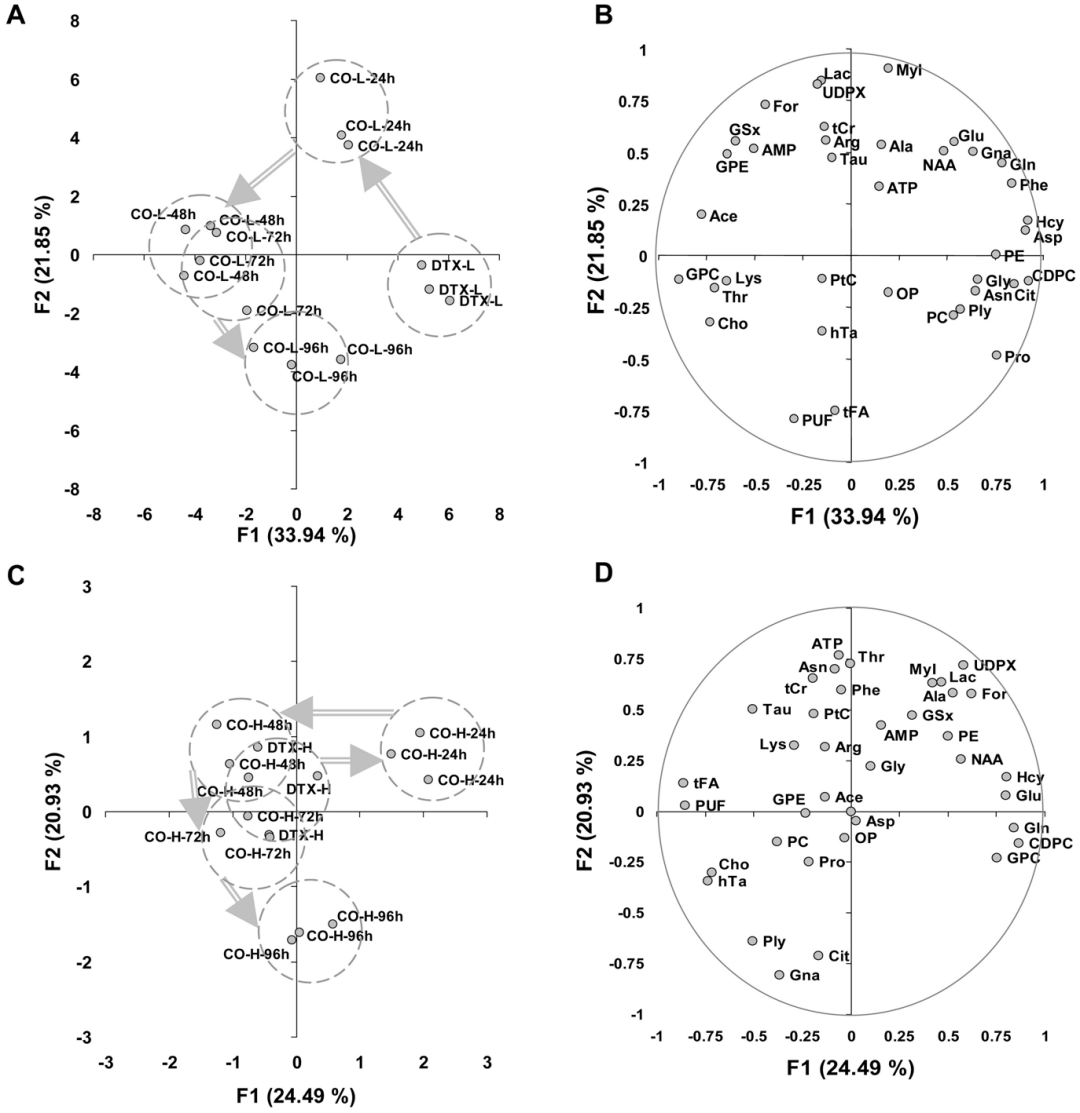
Multivariate analysis of cotreatment-related metabolite data. A- PCA of data related to cotreatment with curcumin and DTX-L. Each score corresponds to an individual measurement and is labeled as in Fig. 5A. F1 and F2, first 2 principal components with the percentage of total variance of data they account for. Cloud contours are displayed for DTX-L, CO-L-24 h, the set of CO-L-48 h and CO-L-72 h, and CO-L-96 h. Arrows, duration of exposure-related trajectory. B- Corresponding loading plot. Metabolite abbreviations, as in Table 1. C- PCA of data related to cotreatment with curcumin and DTX-H. Each score corresponds to an individual measurement and is labeled as in Fig. 5A. F1 and F2, first 2 principal components with the percentage of total variance of data they account for. Cloud contours are displayed for CO-H-24 h, the set of DTX-H, CO-H-48 h and CO-H-72 h, and CO-H-96 h. Grey arrows, duration of exposure-related trajectory. D- Corresponding loading plot. Metabolite abbreviations, as in Table 1.

PCA of cotreatment with DTX-L revealed several separated clouds (Fig. 6A), a first cloud corresponding to samples treated with DTX-L alone, to the right, another cloud corresponding to CO-L-24 h samples, then a cluster of CO-L-48 h and CO-L-72 h samples and a cloud of CO-L-96 h samples. The F1 axis separated the DTX-L group (to the right) alone from co-treatments (to the left). It was explained positively by GSx-related derivatives (Hcy and Asp) and lipid metabolism related-derivatives (CDPC and Cit), and negatively by lipid derivatives (GPC, Cho, Ace). The F2 axis was explained positively by glucose utilization derivatives (Lac, MyI and UDPX), and negatively by lipid metabolism derivatives (tFA and PUF) (Fig. 6B). The metabolic fingerprint underpinned by axis F2 had biphasic or hormetic behavior as a function of duration of co-treatment, with a positive variation at 24 h cotreatment, reverted at 48 h then negative at 96 h.

PCA of cotreatment with DTX-H revealed a cloud of CO-H-24 h samples clearly separated from the others along the F1 axis (Fig. 6C). DTX-H scores in great part superimposed with CO-H-48 h and CO-H-72 h scores. Another cloud was that of CO-H-96 h samples, was well separated along the F2 axis. A metabolic transition was revealed between DTX-H and CO-H-24 h samples, and was fully reverted for 48 h of cotreatment. Axis F1 was mostly explained positively by GSx metabolism derivatives (Glu, Gln, and Hcy) and lipid metabolism derivatives (CDPC, GPC), and negatively by lipid metabolism derivatives (tFA, PUF, Cho) and hTa (Fig. 6D). Axis F2 was mostly explained positively by ATP and negatively by Cit and Gna.

## Discussion

This article reports, to our knowledge, the first metabolomics investigation of cancer cell response to CUR. ^1^H-NMR spectroscopy-based metabolomics was applied to MCF7 and MDA-MB-231 breast cancer cells responding to CUR according to dose, and MCF7 breast cancer cells according to duration of CUR treatment, alone and in combination with DTX. Metabolomics identified prominent targets of CUR in both breast tumor cell lines, including glutathione and lipid metabolism. Multivariate statistical analyses revealed metabolic transitions, even biphasic or hormetic responses with dose and duration of cotreatment with DTX.

### Responses of Breast Cancer Cells to CUR Dose

A major effect of CUR is its ability to both modulate and generate intracellular ROS, as reported in several tumor cell types [1], [15], [17], [20], [29]. ROS in low amounts are implicated in regular cell function, signaling pathways, response to environmental stresses and carcinogenesis. In high amount, ROS cause DNA damage, lipid and protein peroxidation, and apoptosis. In this study, ROS balance between production and quenching could be evaluated from tail DNA measurement which accounts for DNA oxidative damage and effectiveness of DNA repair systems. Tail DNA increased at high doses, in favor that ROS production overwhelmed cellular quenching capacities at those doses. Also, some increase in ROS-related damages was found during delayed (96 h) exposure to CUR used both alone and in combination with DTX. Among metabolites testifying DNA damage, 8-hydroxydeoxyguanosine is the most abundant and by far the most studied. But this biomarker of oxidative stress, as well as others including malonyldialdehyde, a product of lipid peroxidation, are beyond detectability in most NMR-based studies. However, in this study, an unusual product of glucose oxidation significantly increased at 25 mg.l^−1^. This metabolite, Gna, has been reported to accumulate in breast cancer cells in a model of alkaloid-induced oxidative stress [27]. Also, in this study, MCF7 and MDA-MB-231 breast cancer cells demonstrated the same major dose-dependent metabolic targets including glutathione metabolism and lipid metabolism. Because these cell types differ by their expression of hormonal receptors (only MCF7 cells express these receptors, and are sensitive to hormonotherapy), it may be concluded that the reported major metabolic targets do not depend on hormonal signalling pathways, which make CUR a candidate adjuvant therapy for estrogen-negative breast tumors with lower prognosis.

A prominent target of CUR is GSH metabolism, as revealed by GSx and GSx-related magnitude of changes. The main regulatory enzyme of GSH synthesis is glutamate-cysteine ligase (GCL) (Fig. 2A). GSH plays a central role in cell protection against oxidative stress as a cofactor of GSH peroxidases and GST. GST enzymes belong to a family of multifunctional detoxification proteins that protect cells from electrophilic compounds. Overexpression of GST in cancer is implicated in multidrug resistance. However, intense conjugation and efflux may provoke GSH cellular depletion, impairment of detoxification, and cell death. In this study, changes in GSx and related metabolites testified of upregulated biosynthesis of GSH through activation of GCL at low doses explaining the enrolment of transsulfuration (decreased levels of Hcy). Reactivation of GST at 50 mg.l^−1^, at least in part, explained the drop of GSx levels, since both parameters correlate negatively and since biosynthetic activity of GSx was sustained (exhaustion in GSx prcursors). High levels of GSH have been reported in response to CUR [30], [31], as well as upregulation of GCL [30], [31], [32]. Another enzyme, cystathionine beta-synthase (CBS), which regulates transsulfuration, plays an important role in glutathione homeostasis. Our measurements, especially Hcy, show that CBS pathway was recruited to respond to dramatic requirement for GSH. GST activity was biphasic with decreased activity at low dose CUR then increased activity at high dose, in agreement with literature data [33]. Overall, at high dose CUR, increased tail DNA, GST re-activation, GSx decrease, consistent changes in GSx-related metabolites, and Gna increase, together support the fact that ROS production overwhelmed tumor cell defences explaining the drop in DNA content, cell cycle arrest and cell death.

GSH metabolism is regulated by the Nrf2/antioxidant response pathway (ARE) signaling pathway [34], [35]. Nrf2 is a transcription factor that induces genes which promoter contains ARE, including antioxidant enzymes (GCL, glutathione reductase, catalase, glutathione peroxidase, thioredoxin and thioredoxin reductase, NADPH oxidoreductase, heme oxygenase) and phase-2 proteins of the detoxification system (GST, UDP-glucuronosyltransferase) [35]. Nrf2 activation has been implicated in tumor cell multidrug resistance. However, at low dose, CUR inhibited GST activity because of direct inhibition of the enzyme [33], not Nrf2 activity. Our data are consistent with CUR dose-dependent activation of the Nrf2 pathway since they show activation of GCL at low dose then activation of GST at high dose resulting in biphasic behavior of GSx.

Another recently reported enzymatic target of CUR is glyoxylase-1 (GLO1), which links glycolysis to GSH metabolism through the non enzymatic and permanent formation of methylglyoxal, a side product of glycolysis [36]. Methylglyoxal is a toxic 2-oxoaldehyde implicated in ROS production and induction of apoptosis. The simultaneous decrease of Lac and GSx levels at 50 µM curcumin in this study may be partly related to inhibition of GLO1, whereas, at low dose CUR, GLO1 activity should be unaffected. Inhibitors of GLO1 have been proposed as potential anti-carcinogenic agents.

Besides GSH metabolism, another major response involved lipid and phospholipid metabolism. At low dose CUR, there were transient changes in PE and PC, two phospholipid derivatives of ethanolamine and Cho metabolism, respectively. At high dose, there was dramatic increase of tFA and PUF, to which could be related increase of Ace, and decrease of GPC and GPE, two by-products of phospholipid metabolism originating from activity of phospholipase A_2_ (PLA_2_). PLA_2_ products (free fatty acids and lysophosphatidylcholine) are bioactive and can initiate signalling cascades that modulate cellular viability and inflammation. Lysophosphatidylcholine is further catabolized in GPC. Increased fatty acid NMR signals have been shown during accumulation of mobile lipids and onset of apoptosis [37]. Mobile lipid accumulation was reported in cytotoxic insults with inhibitors of mitochondrial respiration involving mitochondrial damage, autophagic vacuoles and lysosomal lipid catabolism [38]. The formation of lipid droplets is another mechanism by which tFA and PUF levels could increase. Lipid droplets are formed when cells can no longer oxidize fatty acids because of deficient mitochondrial functions. This mechanism is considered to prevent lipotoxicity and induction of apoptosis due to accumulating free fatty acids from either exogenous or endogenous sources. Taken together, free fatty acid accumulation correlates with mitochondrial damage. In our study, decrease of GPC and GPE was in favor of downregulation of PLA_2_ activity, possibly as a means for the cell to limit release in the cytosol of membrane-originating fatty acids and propagation of oxidative stress. Cyclooxygenase-2 (COX-2) can be induced by mitogenic and inflammatory stimuli, a mechanism which results in enhanced synthesis of prostaglandins in inflamed and neoplastic tissues. In our study, the accumulation of PUF and tFA at high dose CUR is consistent with inhibition of COX-2. Curcumin was shown to downregulate expression and activity of COX-2 [39] and PLA_2_ [14], [40] both *in vitro* and *in vivo*. It may be proposed that increased levels of PUF and tFA and decreased levels of GPC and GPE indicate inhibition of COX-2 and PLA_2_, both with anti-inflammatory effect. As a matter of comparison, in serum of patients with active tuberculosis, lower levels of lysophosphatidylcholine, the precursor of GPC, were found and attributed to an anti-inflammatory response [41]. Thus our lipid metabolism derivatives are candidate biomarkers of the inflammation status.

Lipid metabolism alterations that we observe at high dose CUR may be linked to NF-κB pathway. NF-κB is a transcription factor that regulates the transcription of many genes for immune response (interleukin-6), cell adhesion (matrix metallopeptidase-9), differentiation, proliferation (COX-2, cyclins), angiogenesis (VEGF), and antiapoptosis (Bcl-2) [42]. NF-κB activation is involved in multiple human pathologies including inflammatory diseases, immune deficiencies, diabetes, and atherosclerosis as well as oncogenesis. CUR has been reported to inhibit NF-κB, thus suppressing various cell survival and proliferative genes, resulting in anti-inflammatory effects [9] [31], [32]. Therefore, at high dose, observed changes in tFA, PUF, GPC and GPE may indicate NF-κB inhibition.

### Responses to CUR in Cotreatment with DTX

The combination of CUR with other anticancer agents deserved much interest, as a mean of sensitizing tumor cells to chemotherapy or other aims. CUR was shown to exert additive effects when combined with vinblastine, a microtubule depolymerizing drug, although antagonistic effects in combination with paclitaxel [21]. Also, CUR at low dose (≤10 µM) inhibited chemotherapy-induced apoptosis in breast cancer cells [20]. Therefore, anticipating the results of a therapeutic association including CUR may reveal at risk, and more investigations are required to better understand the mechanisms of the interaction.

DTX is a taxoid cytotoxic agent that stabilizes microtubule assembly and prevents microtubule depolymerisation, leading to cell cycle arrest in the G2/M phase and apoptosis. Despite CUR and DTX seem to have antagonist effect on microtubules [21], both agents induce cell cycle arrest. In addition, anti-oxidant effects of CUR in noncancerous cells can be exploited as a means of reducing DTX systemic toxicity. This reasoning led to propose the association of CUR with DTX in the treatment of patients with advanced and metastatic breast cancer [25]. The choice of the dose of CUR (10 mg.l^−1^) corresponded to the order of magnitude of concentration that may be reached in human plasma following oral intake [43].

In our study, cotreatment with low dose as well high dose DTX increased DNA content of MCF7 cell pellets when exposure to CUR was 24 h. For longer delays of exposure, DNA content was similar or poorly decreased in comparison to DTX effects alone. Given the above discussion, there is major suspicion that response at 24 h be pro-inflammatory and anti-oxidative. However metabolomics provided further insight into the mechanisms of the response. Combination maintained high levels of GSx whatever the duration of CUR treatment. Thus GSH synthesis and decreased GST activity in response to CUR at 10 mg.l^−1^ were sustained whatever the duration of exposure to CUR. At low dose DTX, GSH synthesis was supported by reduction in Glu (and related Asp) levels, and by active transsulfuration (attested by low Hcy levels). High GSx level could also be sustained by high GLO1 activity. Moreover, tFA and PUF, two of our biomarkers of inflammation, increased at 96 h only, in favor of the emergence of anti-inflammatory effects with long delays of treatment by CUR. Consistently, GPC and GPE which were increased at 48 h and 72 h, in favor of pro-inflammatory effects, tended to decrease at 96 h only. At high dose DTX, tFA and PUF from 24 h to 96 h indicating a sustained pro-inflammatory response due to co-treatment by CUR Consistently, GPC and GPE were between 24 h and 96 h, also in favor of a pro-inflammatory response. Finally, at 72 h and 96 h, accumulated, Gna, an abnormal product of glucose utilization incrased which may announce the emergence of oxidative stress [27], and correlates with other abnormalities in glucose utilization at 96 h (Lac decrease).

Overall, metabolomics draws attention on the fact that associating CUR at *in vivo*-achievable dose to DTX may induce undesirable responses in breast cancer cells including pro-inflammatory and anti-oxidant effects. Therefore the combination needs further evaluation in terms of benefit-toxicity ratio because, although it may alleviate chemotherapy-related toxicity in normal cells, it may reduce chemotherapy efficacy in tumor cells.

### Hormetic Responses to CUR

Some metabolites or metabolite subsets were found to behave biphasically, even hormetically, with dose or duration of association with DTX. Hormetic effects have been reported for cell proliferation with CUR dose [9]. Also, hormetic effects have been described in response to CUR for metabolites or enzyme activities including GLO1 [36], thioredoxin reductase [13] and others [44]. In addition, the disparity of GSH level and GST activity encountered at a single dose of CUR in various (tumoral and non tumoral) cell types may be explained by hormetic behavior [29]. Molecular pathways involved in hormetic response to stressing agents in tumor cells include p53-dependent apoptotic pathways, sphingomyelin metabolism pathway, Nrf2 transcription factor pathway, and others [44].

In cotreatments of CUR with low dose DTX, metabolomics revealed a hormetic component with duration of exposure to CUR, the F2 axis of PCA, which was explained by accumulation of glucose metabolization products (MyI, Lac, UDPX) and diminished lipid content (tFA, PUF). These alterations at 24 h cotreatment, reverted from 48 h to 96 h, thus following a hormetic behavior. CUR was reported to activate the AMPK pathway [40] The involvement of AMPK signaling could account for simultaneous increase in glycolysis (Lac high) and decreased fatty acid biosynthesis, or increased fatty acid catabolism (tFA, PUF low). The second phase of hormesis could take place when AMPK activation was followed by COX-2 inhibition [40], yielding to accumulation of tFA and PUF. Another mechanism which could explain hormesis of metabolic pathways underlain by axis F2 is the response of pyruvate dehydrogenase (PDH) to regulation of PDH kinase by ROS. Metabolite changes similar to those explaing F2 were reported in PDH kinase modulation by ROS [45]. PDH blockade through ROS quenching by CUR at 24 h could account for Lac accumulation and fatty acid decrease. Hormetic reponses should contribute to apparently paradoxical responses to CUR. This is the reason why identifying biomarkers of the response to CUR is important. This study establishes that derivatives of glutathione metabolism (especially GSx) and lipid metabolism (tFA, PUF, GPC and GPE) are candidate biomarkers of the impact of CUR on the redox status and the inflammatory status of treated cells, also able to follow biphasic responses to CUR.

### Conclusions

^1^H-NMR spectroscopy-based metabolomics revealed prominent targets of CUR in MCF7 and MDA-MB-231 breast cancer cells, including glutathione metabolism, lipid/phospholipid metabolism, and glucose utilization. Candidate biomarkers of the response to CUR are proposed, able to inform on oxidative and inflammatory status of breast tumor cells. Also, metabolomics demonstrated metabolic biphasic responses related to CUR dose and duration of cotreatment with DTX, a chemotherapy agent. Biphasic behavior or hormesis likely accounts for apparently paradoxical effects reported for CUR used at different doses, in various therapeutic combinations and cell types, including oxidative and inflammatory status. Such behavior, here demonstrated at the metabolome level and probably underlaid by generalized cellular stress responses, challenges the widely accepted beneficial effects of the phytochemical.

## Supporting Information

Table S1 **CUR dose-related data in MDA-MB-231 cells (fold variation** ***vs.*** **24 h controls).** Mean control value is set to 1 while dispersion of control data is maintained. Metabolite abbreviations, see Table 1. SD, standard deviation. *, P<0.025; $, P<0.01, Mann-Whitney test.
(DOC)
