# Supplementary material for: Metabolomics Reveals Metabolic Targets and Biphasic Responses in Breast Cancer Cells Treated by Curcumin Alone and in Association with Docetaxel
Source: PLoS One. 2013 Mar 5;8(3):e57971. doi: 10.1371/journal.pone.0057971 (PMC3589461; doi:10.1371/journal.pone.0057971)
Supplement: Table S1 — CUR dose-related data in MDA-MB-231 cells (fold variation vs. 24 h controls). Mean control value is set to 1 while dispersion of control data is maintained. Metabolite abbreviations, see Table 1. SD, standard deviation. *, P<0.025; $, P<0.01, Mann-Whitney test. (DOC) [file pone.0057971.s001.doc]

**Table S1. CUR dose-related data in MDA-MB-231 cells (fold variation *vs.* 24h controls).**

| **CUR dose (mg.l^-1^)** | | | | | | | | | | |
| --- | --- | --- | --- | --- | --- | --- | --- | --- | --- | --- |
|  | **0** | | **10** | | | **50** | | |  | |
|  | **Mean** | **SD** | | **Mean** | **SD** | | **Mean** | **SD** | |  |
| **n** | **3** | | | **3** | | | **3** | | |  |
| tFA | 1 | 0.16 | | 0.89 | 0.17 | | 7.84 | 1.56$ | |  |
| PUF | 1 | 0.15 | | 1.16 | 0.01 | | 9.55 | 2.44$ | |  |
| Lac | 1 | 0.11 | | 1 | 0.1 | | 0.56 | 0.11* | |  |
| Ace | 1 | 0.3 | | 0.65 | 0.1 | | 1.26 | 0.44 | |  |
| NAA | 1 | 0.15 | | 0.93 | 0.14 | | 0.89 | 0.2 | |  |
| MyI | 1 | 0.94 | | 3.18 | 0.32* | | 0.58 | 0.43 | |  |
| Glu | 1 | 0.02 | | 0.82 | 0.12 | | 0.9 | 0.07 | |  |
| Gln | 1 | 0.08 | | 0.72 | 0.23 | | 2.72 | 0.26$ | |  |
| Pro | 1 | 0.39 | | 1.08 | 0.35 | | 0.58 | 0.17 | |  |
| Asn | 1 | 0.23 | | 0.8 | 0.16 | | 0.97 | 0.3 | |  |
| Asp | 1 | 0.29 | | 0.57 | 0.26 | | 0.27 | 0.06$ | |  |
| Arg | 1 | 0.2 | | 1.09 | 0.25 | | 0.72 | 0.16 | |  |
| Ply | 1 | 0.27 | | 1.01 | 0.05 | | 0.91 | 0.26 | |  |
| Hcy | 1 | 0.48 | | 0.9 | 0.12 | | 0.53 | 0.25 | |  |
| tCr | 1 | 0.19 | | 1.6 | 0.10* | | 0.22 | 0.06$ | |  |
| hTa | 1 | 0.58 | | 1.47 | 0.31 | | 0.35 | 0.21 | |  |
| Tau | 1 | 0.12 | | 1.19 | 0.22 | | 0.69 | 0.12 | |  |
| GSx | 1 | 0.14 | | 1.92 | 0.25$ | | 0.44 | 0.06$ | |  |
| Gly | 1 | 0.12 | | 0.88 | 0.12 | | 2.94 | 0.48$ | |  |
| Ala | 1 | 0.17 | | 0.95 | 0.2 | | 1.45 | 0.2 | |  |
| Lys | 1 | 0.21 | | 1.03 | 0.39 | | 1.29 | 0.43 | |  |
| Phe | 1 | 0.1 | | 0.83 | 0.21 | | 0.96 | 0.13 | |  |
| Thr | 1 | 0.3 | | 1.21 | 0.29 | | 1.12 | 0.39 | |  |
| PE | 1 | 0.07 | | 1.52 | 0.08$ | | 0.82 | 0.06 | |  |
| GPE | 1 | 0.06 | | 1.13 | 0.1 | | 0.38 | 0.04$ | |  |
| Cho | 1 | 0.08 | | 1.07 | 0.04 | | 0.85 | 0.05 | |  |
| PC | 1 | 0.13 | | 0.89 | 0.14 | | 0.72 | 0.11 | |  |
| CDPC | 1 | 0.18 | | 0.82 | 0.21 | | 1.14 | 0.33 | |  |
| PtC | 1 | 0.3 | | 1.42 | 0.09 | | 1.31 | 0.44 | |  |
| GPC | 1 | 0.04 | | 1.01 | 0.06 | | 0.43 | 0.05$ | |  |
| Gna | 1 | 1.23 | | 2.83 | 2.02 | | 1.86 | 1.62 | |  |
| Cit | 1 | 0.03 | | 1.31 | 0.5 | | 1.28 | 0.2 | |  |
| OP | 1 | 0.27 | | 1.94 | 0.45 | | 1.31 | 0.46 | |  |
| ATP | 1 | 0.38 | | 0.99 | 0.13 | | 0.96 | 0.27 | |  |
| For | 1 | 0.31 | | 0.78 | 0.23 | | 0.69 | 0.17 | |  |
| AMP | 1 | 0.44 | | 1.44 | 0.46 | | 0.89 | 0.33 | |  |
| UDPX | 1 | 0.15 | | 1.24 | 0.2 | | 0.8 | 0.08 | |  |

Mean control value is set to 1 while dispersion of control data is maintained. Metabolite abbreviations, see Table 1. SD, standard deviation. *, P < 0.025; $, P < 0.01, Mann-Whitney test.
